# [Corrigendum] 3-Bromopyruvate sensitizes human breast cancer cells to TRAIL-induced apoptosis via the phosphorylated AMPK-mediated upregulation of DR5

**DOI:** 10.3892/or.2026.9061

**Published:** 2026-01-28

**Authors:** Yuzhong Chen, Li Wei, Xiaojing Zhang, Xianfu Liu, Yansong Chen, Song Zhang, Lanzhu Zhou, Qixiang Li, Qiong Pan, Surong Zhao, Hao Liu

Oncol Rep 40: 2435-2444, 2018; DOI: 10.3892/or.2018.6644

Following the publication of the above article and a corrigendum (doi: 10.3892/or.2022.8264) that was published 3 years ago to resolve the issue of two sets of duplicated western blots in Fig. 3, an interested reader drew to the authors' attention that, in Fig. 5b on p. 2441, the western blot data bands shown to represent the GRP78 and Bcl-2 proteins in the MCF-7 group were strikingly similar; furthermore, the β-actin bands shown for the MDA-MB-231 group in Fig. 5c were strikingly similar to the β-actin bands shown in Fig. 2c on p. 2439.

After having re-examined their original data, the authors realized that the figure parts in question were inadvertently assembled erroneously. The revised version of Fig. 5, now featuring the correct data for the GRP78 western blot bands for the MCF-7 group in Fig. 5b and the β-actin bands in Fig. 5c, is shown in the next page. Note that the errors made in assembling this figure did not affect the overall conclusions reported in the paper. All the authors agree with the publication of this corrigendum, and are grateful to the Editor of *Oncology Reports* for granting them the opportunity to publish this; furthermore, they apologize to the readership for any inconvenience caused.

## Figures and Tables

**Figure 5.**
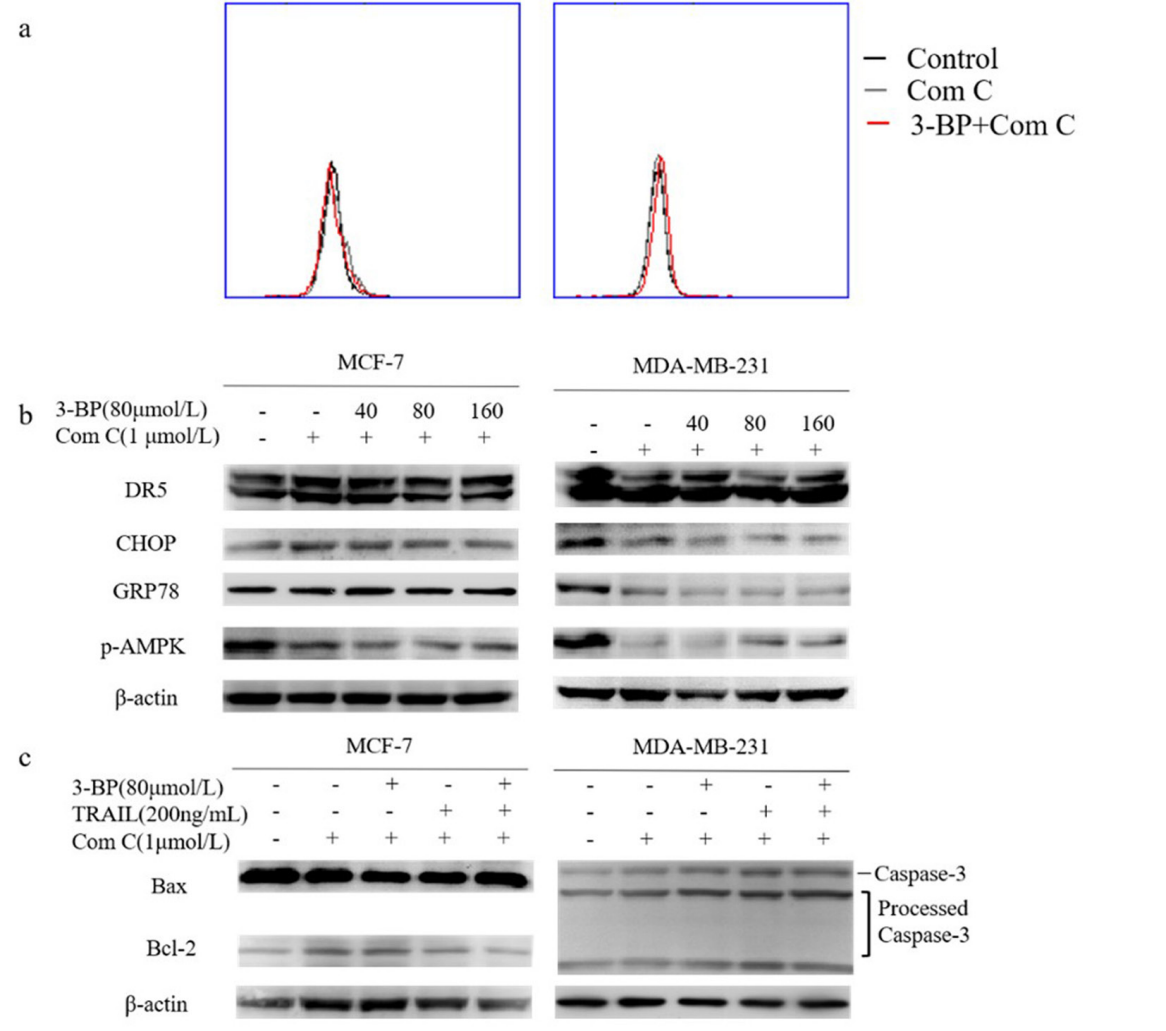
AMPK induces ER stress and sensitizes breast cancer cells to TRAIL in response to treatment with 3-BP. (A) Cells treated with medium (Control), 1 μmol/l Compound C (Com C) or Compound C combined with 80 μmol/l 3-BP for 24 h were investigated via flow cytometry. (B) MCF-7 and MDA-MB-231 cells pre-treated with 1 μmol/l Compound C for 1 h were subsequently treated with 0, 40, 80 or 160 μmol/l 3-BP for 24 h. The expression levels of AMPK, GRP78, CHOP and DR5 were investigated with western blotting. (C) Cells pre-treated with or without 1 μmol/l Compound C for 1 h, were treated with medium, Compound C, 80 μmol/l 3-BP, 200 ng/ml TRAIL or both 3-BP and TRAIL, as indicated, for 24 h. The expression levels of Bax and Bcl-2 were determined in MCF-7 cells and caspase-3 was investigated in the MDA-MB-231 cells by western blotting. β-actin served as loading control. TRAIL, tumor necrosis factor-related apoptosis-inducing ligand; 3-BP, 3-bomopyruvate; DR5, death receptor 5.

